# [Retracted] MicroRNA‑19b promotes the migration and invasion of ovarian cancer cells by inhibiting the PTEN/AKT signaling pathway

**DOI:** 10.3892/ol.2024.14424

**Published:** 2024-04-30

**Authors:** Dan-Tong Liu, Hai-Rong Yao, Yan-Ying Li, Yang-Yang Song, Meng-Ya Su

Oncol Lett 16: 559–565, 2018; DOI: 10.3892/ol.2018.8695

Following the publication of this paper, it was drawn to the Editor's attention by a concerned reader that the Transwell migration and invasion assay data shown in Fig. 3C on p. 563 were strikingly similar to data that had already been published in different form in another article written by different authors at a different research institute [Zhuang H, Tan M, Liu J, Hu Z, Liu D, Gao J, Zhu L and Lin B: Human epididymis protein 4 in association with Annexin II promotes invasion and metastasis of ovarian cancer cells. Mol Cancer 13: 243, 2014].

Owing to the fact that the contentious data in the above article had already been published prior to its submission to *Oncology Letters*, the Editor has decided that this paper should be retracted from the Journal. The authors were asked for an explanation to account for these concerns, but the Editorial Office did not receive a reply. The Editor apologizes to the readership for any inconvenience caused.

